# *β*_3_ Adrenergic Receptor Stimulation Promotes Reperfusion in Ischemic Limbs in a Murine Diabetic Model

**DOI:** 10.3389/fphar.2021.666334

**Published:** 2021-04-22

**Authors:** Kristen J. Bubb, Dhanya Ravindran, Siân P. Cartland, Meghan Finemore, Zoe E. Clayton, Michael Tsang, Owen Tang, Mary M. Kavurma, Sanjay Patel, Gemma A. Figtree

**Affiliations:** ^1^ University of Sydney, Faculty of Medicine and Health, Sydney, NSW, Australia; ^2^ Kolling Institute of Medical Research, Royal North Shore Hospital, St Leonards, NSW, Australia; ^3^ Department of Physiology, Biomedicine Discovery Institute, Faculty of Medicine, Nursing and Health Sciences, Monash University, Clayton, VIC, Australia; ^4^ Heart Research Institute, Eliza St Newtown, Sydney, NSW, Australia

**Keywords:** peripheral artery disease, nitric oxide, redox, hind limb ischemia, vascular

## Abstract

**Aims/Hypothesis:** Peripheral arterial disease (PAD) is a major burden, resulting in limb claudication, repeated surgical interventions and amputation. There is an unmet need for improved medical management of PAD that improves quality of life, maintains activities of daily life and reduces complications. Nitric oxide (NO)/redox balance is a key regulator of angiogenesis. We have previously shown beneficial effects of a *β*_3_ adrenergic receptor (*β*_3_AR) agonist on NO/redox balance. We hypothesized that *β*_3_AR stimulation would have therapeutic potential in PAD by promoting limb angiogenesis.

**Methods:** The effect of the *β*_3_AR agonist CL 316,243 (1–1,000 nmol/L *in vitro*, 1 mg/kg/day *s. c*) was tested in established angiogenesis assays with human endothelial cells and patient-derived endothelial colony forming cells. Post-ischemia reperfusion was determined in streptozotocin and/or high fat diet-induced diabetic and non-diabetic mice *in vivo* using the hind limb ischemia model.

**Results:** CL 316,243 caused accelerated recovery from hind limb ischemia in non-diabetic and type 1 and 2 diabetic mice. Increased eNOS activity and decreased superoxide generation were detected in hind limb ischemia calf muscle from CL 316, 243 treated mice vs. controls. The protective effect of CL 316,243 in diabetic mice was associated with >50% decreases in eNOS glutathionylation and nitrotyrosine levels. The *β*_3_AR agonist directly promoted angiogenesis in endothelial cells *in vitro*. These pro-angiogenic effects were *β*_3_AR and NOS-dependent.

**Conclusion/Interpretation:** *β*_3_AR stimulation increased angiogenesis in diabetic ischemic limbs, with demonstrable improvements in NO/redox balance and angiogenesis elicited by a selective agonist. The orally available *β*_3_AR agonist, Mirabegron, used for overactive bladder syndrome, makes translation to a clinical trial by repurposing of a *β*_3_AR agonist to target PAD immediately feasible.

## Introduction

Atherosclerotic peripheral arterial disease (PAD) affects ∼200 million people world-wide and is particularly prevalent over age 70 (European Stroke et al., 2011). In addition to impacting quality of life, PAD is a strong predictor of cardiovascular (CV) mortality (Norgren et al., 2007). Peripheral limb arterial stenoses limit blood flow and lead to insufficient oxygenation, exacerbated by exertion. Manifestations include intermittent claudication or pain, and subsequent limited mobility (Hamburg and Creager, 2017). The most severe form of PAD is critical limb ischemia, complicated by pain at rest, ulcers and gangrene, and an amputation risk of ∼10–40% at 6 months (Uccioli et al., 2018). The economic impact of PAD is immense with the burden on the health budget >$21 billion in the United States alone (Mahoney et al., 2008). Current approaches are focused on reducing atherosclerosis progression and include smoking cessation, as well as statin, antiplatelet and antihypertensive therapy (European Stroke et al., 2011). However, medical therapies to address ischemia and its clinical consequences are lacking. Although supervised exercise programs show some benefits, they are not always feasible (Hamburg and Creager, 2017). The paucity of medical therapies targeting ischemia has led to many patients resorting to endovascular revascularization procedures to mechanically address atherosclerotic stenoses, yet the mid-long term clinical benefits are disappointing (Salhiyyah et al., 2015). Indeed, repeat attempts at revascularization are often required. There is a clear need for improved pharmacological options for directly targeting the underlying disease mechanisms to improve function and quality of life of PAD patients.

Therapeutic angiogenesis promotes collateral vessel formation and this is a promising strategy for treating PAD (Iyer and Annex, 2017). A range of candidates to stimulate angiogenesis in PAD have been identified, but none have been successful in translation to clinical practice and the search for superior novel targets continues. Vascular nitric oxide (NO) production is reduced in patients with PAD (Gerhard-Herman et al., 2006) and targeting nitric oxide signaling has potential value (Steven et al., 2017) due to the vital role of NO in vascular function, angiogenesis as well as protection against progression of atherosclerosis itself (Kenjale et al., 1985; Lerman and Zeiher, 2005; Steven et al., 2017). Uncoupling of endothelial NO synthase (eNOS) occurs early in the process of atherosclerosis. With this, a dramatic switch occurs whereby the free radical, superoxide is produced at the expense of NO (Brown and Griendling, 2015; Karimi Galougahi et al., 2015). Therefore, therapies aimed at protecting against redox-dependent eNOS uncoupling have the potential to enhance angiogenesis and be of potential benefit for patients with ischemic symptoms and complications.

Like their better known family members *β*_1_ and *β*_2_ adrenergic receptors (ARs), *β*_3_ARs have an important functional role in the cardiovascular system (Gauthier et al., 2000; Balligand, 2016; Cannavo and Koch, 2017). *β*_3_AR -agonist signaling is Gi⍺-receptor-mediated (Cannavo and Koch, 2017). *β*_3_AR agonists produce peripheral vasodilation (Berlan et al., 1994; Shen et al., 1994; Shen et al., 1996) attributable to eNOS activation and NO release (Bundgaard et al., 2010; Galougahi et al., 2012), and cyclic guanosine monophosphate (Cannavo and Koch, 2017). Endothelial dysfunction can be rescued by a *β*_3_AR agonist due to improved NO/redox balance *in vivo* (Karimi Galougahi et al., 2016). Studies from over a decade ago have shown *β*_3_AR stimulation increases angiogenesis in retinal (Steinle et al., 2003) and coronary (Dessy et al., 2005) vascular endothelial cells, yet the therapeutic potential of this has not been assessed. We hypothesized that *β*_3_ARs would modulate limb angiogenesis and prove useful as a treatment for PAD. In the current study we examine the efficacy of *β*_3_AR stimulation on NO/redox balance in the regulation of angiogenesis. We also determined the impact on lower limb perfusion in preclinical models of PAD and diabetes.

## Methods

All study drugs were obtained from Sigma Aldrich, Australia, unless otherwise stated. All animal and human studies conform to international standards, and were approved locally as detailed in the relevant sections.

### Endothelial Cell Culture

Human umbilical vein endothelial cells (HUVECs; Lonza C2519AS, pooled source, Australia) were grown using standard cell culture conditions in endothelial cell growth medium (EGM plus^®^, containing 2% fetal bovine serum, Lonza, Australia). All cells were regularly confirmed to be *mycoplasma* negative. Two different pooled source cell lines were used in experiments and all were used within passages 2–4. Human adult dermal microvascular endothelial cells were also obtained from Lonza (CC-2543, Lonza Australia) and cultured as above but using endothelial growth medium 2-MV bulletkit. Endothelial colony forming cells (ECFCs) were derived from the peripheral blood of participants in the BioHEART study (Kott et al., 2019). This registered study (ACTRN12618001322224) complies with the Declaration of Helsinki and the study protocol and design has been approved by the Northern Sydney Local Health District Human Research Ethics Committee (HREC/17/HAWKE/343). Peripheral blood samples were collected from participants following insertion of a venous cannula for a clinically indicated CT coronary angiogram (CTCA). Coronary artery disease status was obtained from coronary calcification assessment using Gensini scoring as outlined previously (Kott et al., 2019). Blood was transferred into lithium heparin pathology tubes and stored at room temperature. Peripheral blood mononuclear cells (PBMCs) were isolated within 4 h of blood collection using a standard gradient-separation Ficoll preparation (Riedhammer et al., 2016). Briefly, PBMCs were plated into 0.1% gelatin-coated flasks at a density of 2.5 × 10^4^ cells/cm^2^ in endothelial cell growth medium containing 2% fetal bovine serum (EGM2 bulletkit, Lonza, Australia). The flasks were cultured in standard conditions for up to 21 days, with regular monitoring for spontaneous growth of ECFCs. Individual cell lines were frozen down in FBS with 10% DMSO and stored in liquid nitrogen. Selected cell lines based on participants coronary artery disease status were thawed for use in tubule formation and the associated health data was extracted from the biobank database.

### Tubule Formation and Cell Migration

Once they were ∼70% confluent, cells were re-suspended in diluted EGM plus^®^ (1:3) and plated on reduced-growth factor extracellular matrix (15 mg/ml, Cultrex, Trevigen, United States) at a density of 1.5 × 10^4^ cells/cm (Norgren et al., 2007). Cells were treated with *β*_3_AR agonist, CL 316,243 at concentrations ranging from 1 to 1,000 ng/ml. Some experiments were conducted in the presence of non-selective NOS inhibitor, Nω-nitro-l-arginine methyl ester (l-NAME, 300 μmol/L) or selective *β*_3_AR antagonist SR 59230A (1 μmol/L). Cells were incubated in an EVOS FL Auto Imaging system for 16 h and tubule formation recorded every hour. Optimal tube formation occurred at the 8-h timepoint; tubule number at this point was quantified manually using NIH ImageJ software. For migration studies, HUVECs were plated in a 96 well plate at a density of 6 × 10^5^ cells/cm^2^ in EGM plus and left to reach confluence. A scratch was performed using a 10 μL sterile pipette and media was replaced with diluted EGM plus^®^ (1:3, as above). Cells were treated with CL 316,243 at concentrations ranging from 1 to 1,000 ng/ml and images were taken at 3-hourly intervals over a 48-h period.

### Murine Hind Limb Ischemia Model

All animal procedures were approved by the Sydney Local Health District Animal Ethics committee (approval number 2016/007) and conform to the National Health and Medical Research Council of Australia’s Code of Practice for the Care and Use of Animals for Scientific Purposes.

Male C57BL6/J mice 8–10 weeks of age were obtained from Australian BioResources (Moss Vale, NSW) with 12 h light/dark cycles and free access to water and mouse chow (Specialty Feeds, Australia). Mice were housed in groups of 2-5 in standard cages within a Physical Containment Level 2 laboratory. For *in vivo* angiogenesis 16 mice underwent the femoral vascular ligation model. Mice were anesthetized with 1.5–2% isoflurane vaporized in oxygen and constant body temperature was maintained. All mice received pre-operative and 24-h post-operative analgesia (carprofen, 5 mg/kg s. c). A small incision (∼15 mm) was made in the hind limb skin directly over the femoral vasculature. A portion of the femoral artery and vein, distal to the origin of the *profunda femoris* artery and proximal to the saphenous artery, were isolated and two ligations were performed using 6–0 silk sutures. The femoral artery and vein were then excised between the ligation sites (Clayton et al., 2017). An osmotic mini-pump (model 1,002 [for 14 days protocols] or model 1,004 [for 28 days protocols], Alzet, United States) containing either CL 316,243 (1 mg/kg/day) or vehicle (normal saline) was then implanted via the femoral skin opening and tunneled around and positioned in the dorsal flank. The mice were randomized 1:1 to treatment protocols prior to undergoing surgery. The skin was closed with non-continuous suture (Prolene 6–0, Johnson and Johnson Medical, Australia). Hind limb blood flow was assessed prior to ligation (baseline) and immediately after undergoing hind limb ischemia, with subsequent imaging at day 3, 7, 10, 14, 21 and 28 post hind limb ischemia. Some experiments were stopped at 14 days for assessment of hind limb histology and biochemistry. Software was used to analyze the perfusion flux (Moor, United Kingdom) and investigators were blinded to the treatment groups during the period of analysis. The rate of reperfusion in the hind limb was calculated as a ratio of blood flow in the ischemic vs. non-ischemic contralateral limb.

Type 1 diabetes model: 20 C57BL6/J mice 6–8 weeks of age were injected with streptozotocin on 5 consecutive days (55 mg/kg, i. p) to induce pancreatic islet destruction with subsequent hyperglycemia as described previously (Prakoso et al., 2017). 16 Non-diabetic control mice received vehicle injections (0.1 mol/L sodium-citrate buffer, pH 4.5, i. p). Mice were monitored weekly and blood glucose was measured using a handheld glucometer (Roche Accu-chek) with a blood sample obtained via tail prick. Four weeks after the last injection, mice were randomized (1:1) to receive CL 316,243 or vehicle treatment and underwent hind limb ischemia and minipump implantation as described above. Following randomization 1 control mouse allocated to vehicle treatment died during a procedure due to equipment failure and 1 diabetic mouse randomized to the CL 316,243 group did not recover from surgery.

Type 2 diabetes model: 30 C57BL6/J mice at 6 weeks of age were injected with streptozotocin on 3 consecutive days (55 mg/kg i. p) and concurrently transitioned onto a high fat diet (Tate et al., 2019) (42% energy intake from lipids, SF04–001, Specialty Feeds, Australia). 24 Non-diabetic time-matched controls were injected with citrate buffer vehicle and fed standard rodent chow. Mice were kept for 20 weeks on high fat diet prior to undergoing hind limb ischemia as described above. Mice were randomized 1:1 to receive CL 316,243 or saline vehicle and this was implanted during hind limb ischemia surgery as outlined above. Following randomization 1 diabetic mouse, allocated to CL 316,243, died during surgery and 1 control mouse, allocated to CL 316,243, did not recover from surgery. 4 diabetic mice (2 per group) were excluded from the study due to blood glucose levels dropping below 15 nmol/L.

Glucose tolerance testing was conducted in fasted type 2 diabetic mice. Rodent chow was removed overnight and testing was conducted in the morning. After baseline glucose testing mice were injected intraperitoneal with sterile D-glucose (2 g/kg). Repeated blood glucose sampling was conducted every 15–30 min for 2 h.

### Histological Analysis

Formalin fixed paraffin-embedded gastrocnemius was cut into 4 µm sections and then sections were deparaffinized. Heat retrieval was performed with Tris-EDTA buffer at pH 9. Slides were incubated overnight with a rabbit polyclonal CD31 antibody (dilution 1:200, Abcam Ltd., Australia) followed by horseradish peroxidase anti-rabbit Envision system (Dako Cytochemistry, Tokyo, Japan). Staining was developed with 3.3 diaminobenzidine tetrahydrochloride (Dako Cytochemistry, Tokyo, Japan) and counterstained with Mayer’s hematoxylin stain. Rabbit IgG negative controls (Dako Cytochemistry, Tokyo, Japan) were used. A total of ten non-overlapping images for each gastrocnemius were taken with a light microscope (Leica, DM750 linked to an ICC50 E camera module). Images were taken at x40 and analyzed with National Institute of Health ImageJ 1.51j8 software.

### Biochemical Analysis

Hind limb tissue including the gastrocnemius and adductor muscles were isolated and collected at 14 or 28 days. Tissues were separated and implanted in OCT or placed in cryovials and snap-frozen in liquid nitrogen or were fixed in 10% formalin for 24-h and then moved to 70% ethanol for storage.

### Superoxide Anion Generation

Frozen adductor tissue was prepared for lucigenin-enhanced chemiluminescence assay by homogenizing in lysis buffer (250 mM sucrose in phosphate-buffered saline (mM: 129 NaCl, 7 Na_2_HPO_4_, 3 NaH_2_PO_4_.2H_2_O, pH 7.4, with protease inhibitors (cOmplete™ EDTA-free, Roche Diagnostics). Sample was added to opaque 96-well plates in the presence of lucigenin (20 μmol/l N,N′-Dimethyl-9,9′-biacridinium dinitrate) and NADPH (100 μM; *β*-Nicotinamide adenine dinucleotide 2′-phosphate reduced tetrasodium salt hydrate). The reaction was conducted at room temperature and tracked using a luminometer (Veritas, Turner Biosystems, United States) with an average measurement taken from 20 cycles, as described previously (Bubb et al., 2018). Replicates of each sample were treated with manganese TMPyP (Merck Millipore, Australia, a cell-permeable superoxide dismutase mimetic, 30 μmol/L), during the assay and any signal was subtracted from the total signal as non-superoxide background signal. Superoxide production was normalized to protein concentration or cell count.

### NO Synthase Activity

The activity of NOS was measured using radioimmunoassay according to manufacturer’s instructions (Cayman Chemical, United States). Samples were prepared in triplicate and detected using a liquid scintillation counter (5 min detection, Tri-Carb 4910 TR 100 V, Perkin Elmer, United States). All samples were also assayed in the presence of l-NAME and this was subtracted from the baseline to give a readout of NOS activity.

### Immunoblotting

Gastrocnemius samples were stored at −80^°^C and then mechanically homogenized in ice-cold lysis buffer containing 150 mmol/L NaCl, 200 mmol/L Tris-HCl (pH 8.0), 1% Triton X-100, 0.5% deoxycholic acid, 0.1% SDS, N-ethylmaleimide (25 mM) and protease inhibitors (cOmplete™ EDTA-free, Roche Diagnostics). 30 μg of protein lysate was denatured and run under reducing conditions on SDS-PAGE (Bolt™ pre-cast gels and reagents, Thermofisher Scientific, Australia) and transferred onto Immobilon polyvinylidene fluoride membrane (Merck Millipore, Australia). Membranes were incubated in primary antibodies directed at determining protein expression of the following: Nox isoforms (anti-Nox 2, 1:5,000; Abcam, Australia; anti-Nox-4, 1:5,000; Abcam, Australia); reactive nitrogen species (anti-nitrotyrosine, 1:1,000; Abcam, Australia); and both expression and phosphorylation of eNOS (anti Phospho eNOS serine 1,177, 1:1,000, Cell Signaling Technology, United States; anti-eNOS 1:1,000, BD Biosciences, United States) and Akt (anti Phospho Akt 1:1,000, Akt 1:1,000, Cell Signaling Technology, United States). Specific secondary antibodies recognizing rabbit or mouse primary antibodies were used (IRDye^®^, Licor; 1:20,000, United States). Membranes were detected using an Odyssey imaging platform (Licor, United States) (Bubb et al., 2018).

### Immunoprecipitation

Gastronemius protein (500 μg) extracted as above was used for co-immunoprecipitation with eNOS. Protein G dynabeads (1.5 mg/ml, 2.8 μm beads, Thermofisher Scientific, Australia) were covalently conjugated with mouse anti-eNOS antibody (BD Biosciences, 1 μg) using bis(sulfosuccinimidyl) suberate amine-amine cross-linking solution (5 mM; ThermoFisher Scientific, Australia). Beads were washed with PBS and incubated with protein lysate overnight at 4°C. IgG controls were prepared using anti-IgG antibodies conjugated to dynabeads using an identical process. Protein was eluted from beads using LDS buffer, denatured and run in non-reduced conditions on 8% Bis-Tris gels using SDS-PAGE and transferred onto polyvinylidene fluoride membrane as above. Expression of oxidized glutathione was detected using mouse anti-glutathione antibody (Virogen, 1:1,000). eNOS was detected using rabbit anti-eNOS (Cell Signaling Technology, 1:1,000, Australia). Odyssey detection system was used to visualize bands as above.

### Sample Size and Statistical Analysis

Data are expressed as mean ± standard error of the mean (SEM). Student’s t-test was used for comparison between two groups. For multiple comparisons, 1- or 2-way analysis of variance (ANOVA) was used with Bonferroni post-hoc analysis for multiple comparisons. A *p* value < 0.05 was considered statistically significant. For all mouse studies, sample sizes were calculated based on the 80% power to detect a 30% change in primary endpoint (perfusion ratio) with standard deviation of 25%. Additional mice were added to diabetes groups based on variability and failure rates of diabetes models.

## Results

### *β*_3_AR Stimulation Promotes Angiogenesis

**FIGURE 1:**
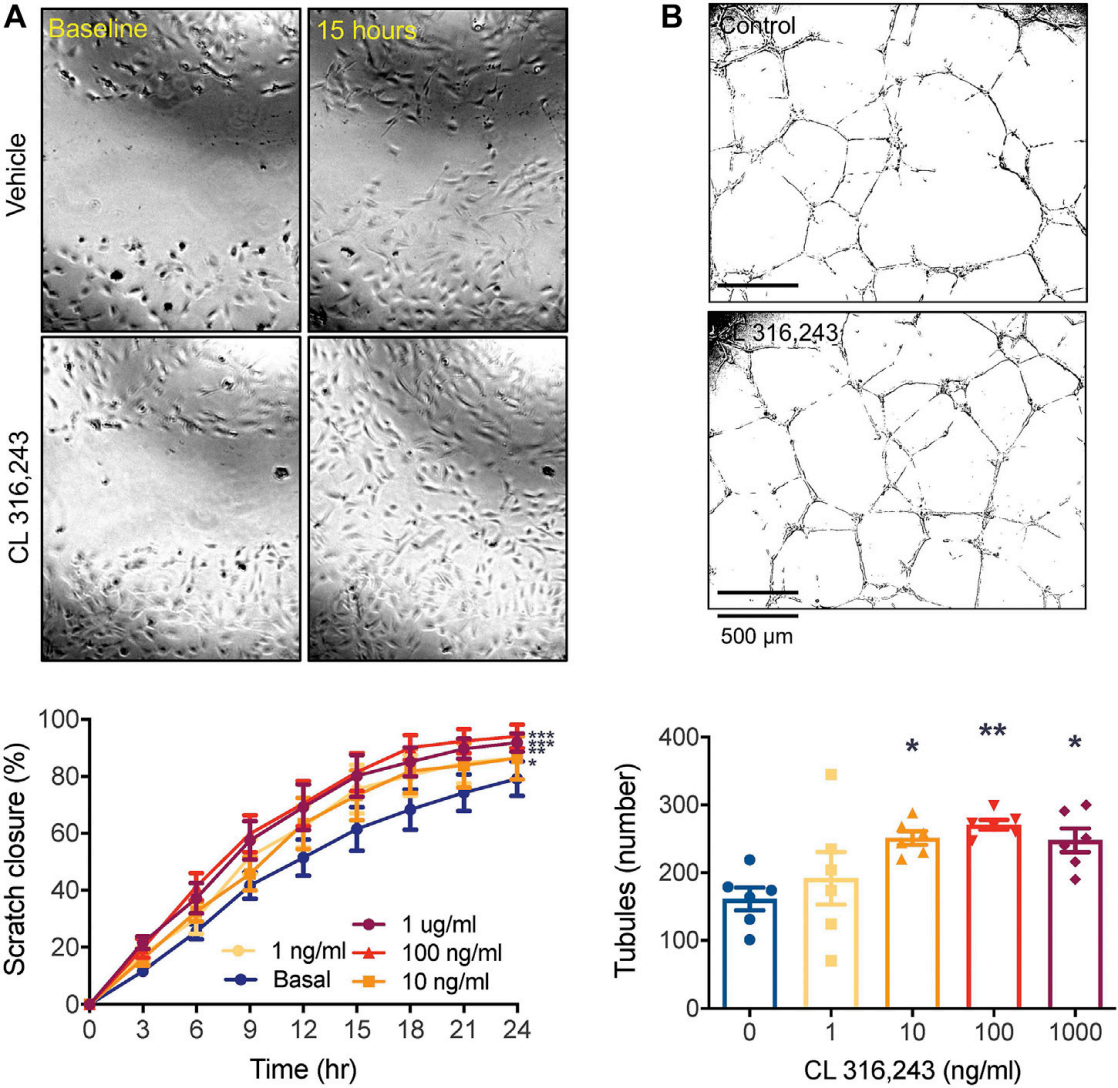
β_3_AR stimulation promotes angiogenesis *in vitro*. **(A)** cell migration by scratch assay over 24 h in response to increasing concentrations of CL 316,243 in HUVECs. ×4 magnification of 96 well plate.; *p < 0.05, **p < 0.01, ***p < 0.001, ****p < 0.0001 vs vehicle control by 2-way ANOVA and **(B)** tubule formation in HUVECs grown on reduced growth factor Cultrex extracellular matrix, in response to increasing concentrations of β3 AR agonist CL 316,243. *p < 0.05, **p < 0.01, vs vehicle control by 1-way ANOVA with Bonferroni post-hoc analysis, *n* = 6 from 3 experiments. All data shown is mean ± SEM. Representative images depict data obtained from vehicle control and CL 316,243-treated (100 ng/ml) cells, with panel A showing closure at baseline (left) and after 15 h (right).

We first established a role for *β*_3_AR stimulation in promoting angiogenesis *in vitro* using HUVECs. The *β*_3_AR agonist, CL 316,243, significantly increased migration of HUVECs into the denuded zone (Figure 1A), with >90% closure reached by 24 h at the higher concentrations. CL 316,243 also increased the number of tubules formed. This was significantly increased by the 10 and 100 ng/ml concentrations compared to the control (Figure 1B).

**FIGURE 2:**
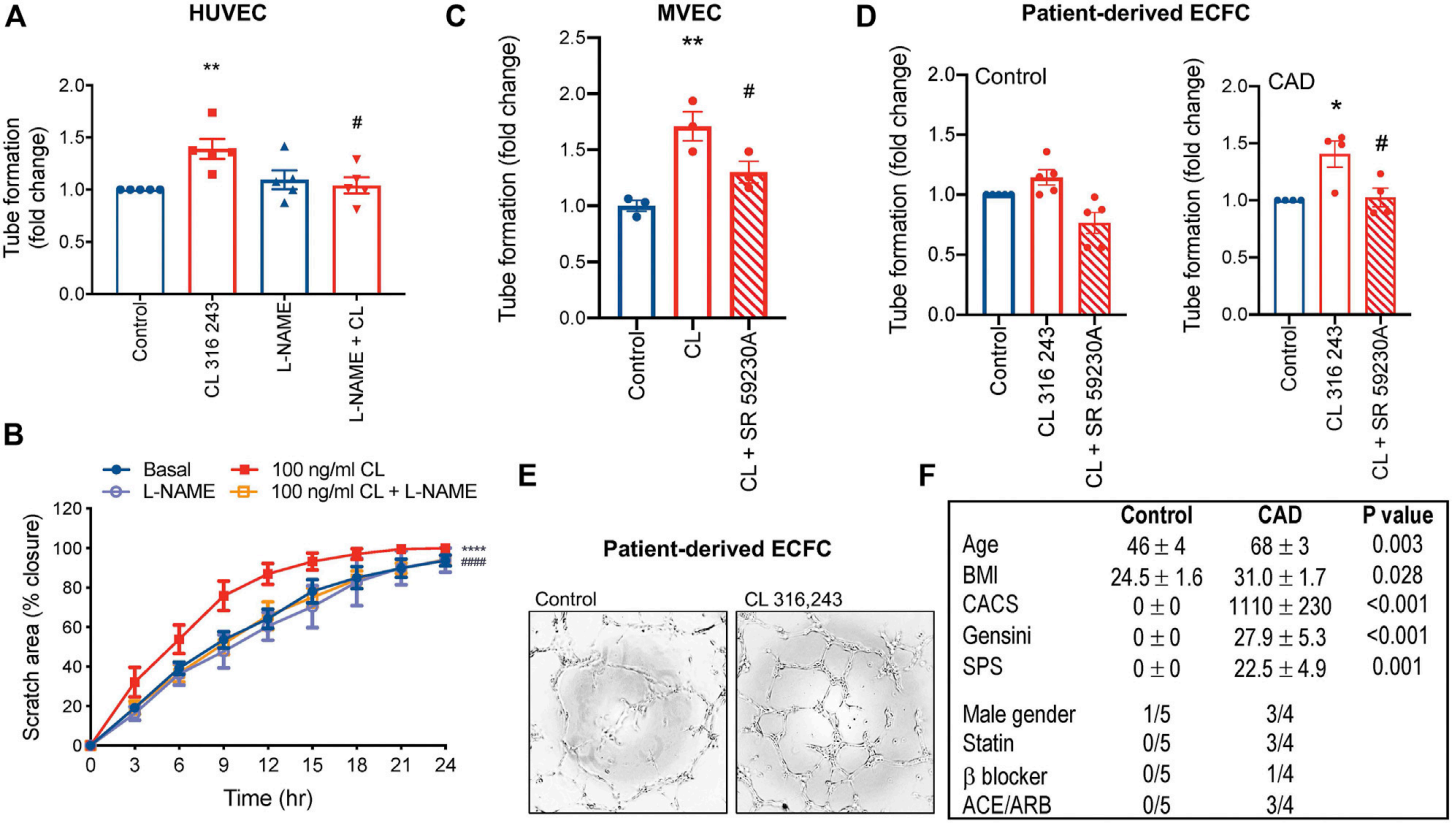
β_3_AR-stimulated angiogenesis is NOS-dependent. **(A)** Tubule formation in HUVECs and the effect of l-NAME (300 μmol/L); *n* = 5 **(B)** cell migration rate in HUVECs and the effect of l-NAME (300 μmol/L); *n* = 4. **(C)** Tubule formation in human adult dermal microvascular endothelial cells (MVEC) and **(D)** patient-derived endothelial colony forming cells (ECFC) with *β*_3_AR agonist CL 316,243 (100 ng/ml) and *β*_3_AR antagonist, SR 592630 A (1 μmol/L) *n* = 3. **(E)** Representative images in ECFCs and **(F)** participant characteristics and medical history of ECFC source. Mean ± SEM; **p < 0.01, ****p < 0.0001 vs control; ^#^ *p* < 0.05, ^##^ *p* < 0.01, ^####^ *p* < 0.0001 vs. CL 316,243 by 1-way ANOVA with Bonferroni post-hoc analysis or 2-way ANOVA. ACE/ARB, angiotensin converting enzyme inhibitor/angiotensin receptor blocker; BMI, body mass index; CACS, coronary artery calcium score; SPS, soft plaque score.

To confirm that *β*_3_AR-inducible angiogenesis involves eNOS activation and NO in our system, we exposed HUVECs to l-NAME. The pro-angiogenic effects of *β*_3_AR were abolished with l-NAME (Figures 2A,B). CL 316,243 also stimulated angiogenesis in endothelial cells from adults (dermal source, Figure 2C), and this effect was *β*_3_AR specific, since CL 316,243-induced tubule formation was abolished in response the *β*_3_AR antagonist, SR 592230 A (Figure 2C). Furthermore, CL 316,243 could also effectively stimulate angiogenesis via *β*_3_ activation in ECFCs derived from patients with significant coronary artery disease (Figures 2D–F). Interestingly, in ECFCs derived from healthy volunteers, the effect was less and did not reach statistical significance (Figure 2D).

### *β*_3_AR Stimulation Accelerates Reperfusion Following Hind Limb Ischemia

**FIGURE 3:**
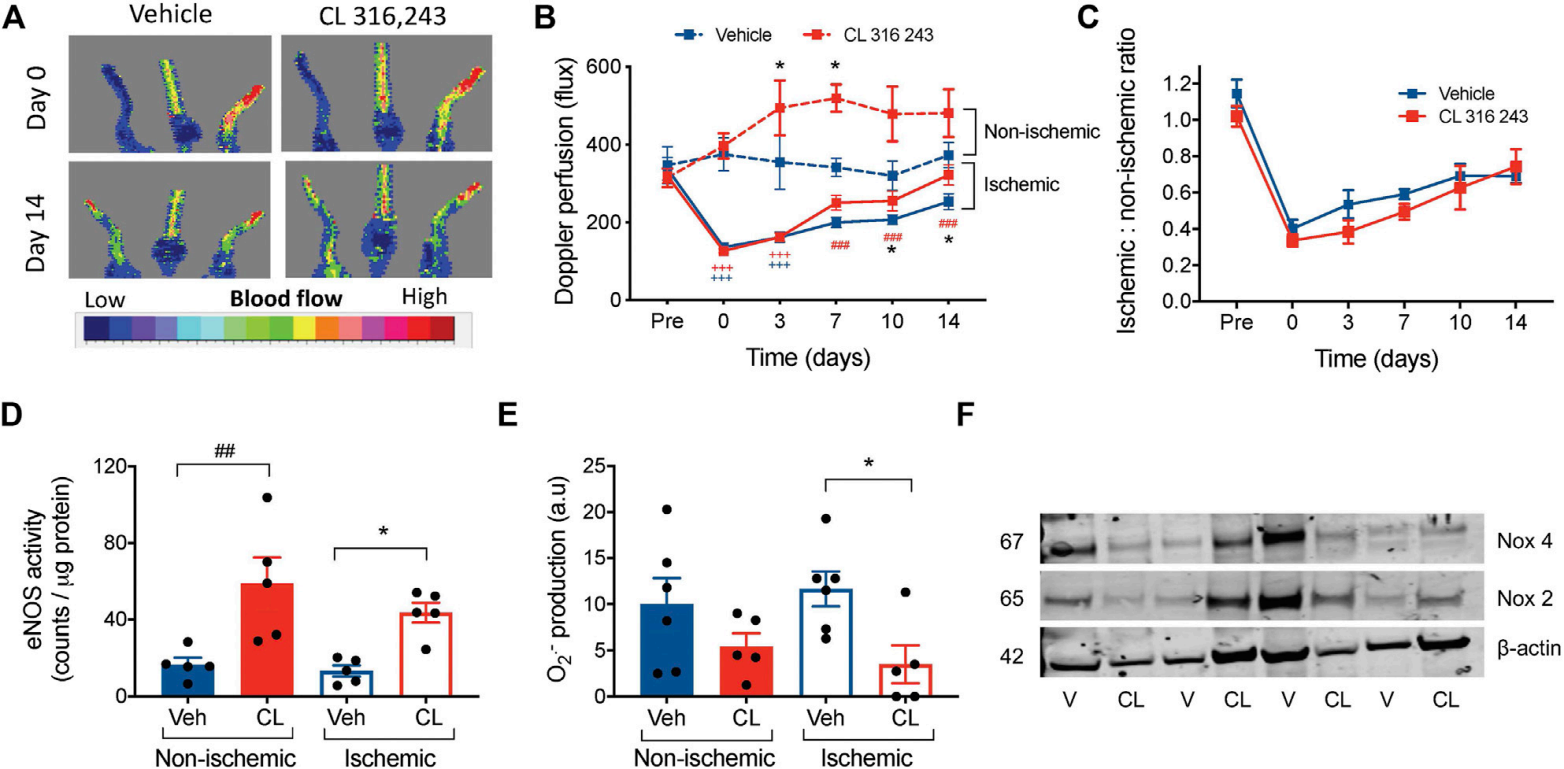
β_3_AR stimulation accelerates reperfusion following hind limb ischemia. **(A)** Representative images showing Laser Doppler flux in hindlimbs from mice immediately post-ligation and after 14 days of recovery, treated with vehicle (saline) or CL 316,243 (1 mg/kg/day) **(B)** Summary data of hind limb perfusion both pre- and post-ligation and in the contralateral control limb shown as raw flux data. **(C)** Calculated ratio of the ischemic to non-ischemic limbs following 14 days of hind limb ischemia. **(D)** eNOS activity by radioimmunoassay in hind limb tissue from mice treated with vehicle or CL 316,243, at 14 days post-surgery. **(E)** Superoxide generation measured by lucigenin-enhanced chemiluminescence (20 μmol/L) corrected for background luminescence. **(F)** Immunoblot expression of hindlimb Nox 2 and Nox 4 with *β*-actin control, V = vehicle, CL = CL-316,243-treated. Mean ± SEM; *p < 0.05 vs vehicle by 1-way or 2-way ANOVA with Bonferroni post-hoc analysis; *n* = 8. ^+++^ *p* < 0.001 vs. Pre-ligation limb perfusion; ^###^ *p* < 0.001 vs. post-ligation (timepoint 0) reperfusion.

We next examined the angiogenic potential of *β*_3_AR *in vivo* in a model of hindlimb ischemia. Ligation of hindlimb vascular beds resulted in severely impaired perfusion compared to pre-ligation in both groups (Figures 3A,B). Although subcutaneous infusion of CL 316, 243, but not vehicle, significantly increased perfusion in the ischemic limbs 10–14 days following ischemic injury, systemic infusion of CL 316,243 also increased perfusion in the non-ischemic limb (Figures 3A,B). When the ischemic-non-ischemic ratio was calculated (Krishna et al., 2020), no differences were observed between vehicle and CL 316 243-treated mice (Figure 3C). We next determined whether CL 316,243 infusion increased eNOS activity in both control and ischemic limbs, thus contributing to the increased perfusion. Indeed CL 316,243 enhanced eNOS activity in both the ischemic and non-ischemic limbs (Figure 3D). Furthermore, superoxide bioavailability in the ischemic limb was significantly lower after 14 days of CL 316,243 infusion (Figure 3E) and not related to expression of NADPH oxidase (Nox) isoforms; no significant differences in hindlimb protein expression for Nox 2 or 4 were observed (Figure 3F, fold change from vehicle in ischemic limb: Nox 2, 1.04 ± 0.46; Nox 4 0.88 ± 0.28, *n* = 4, *p* > 0.05).

Collectively, these findings suggest that *β*_3_AR stimulation can improve post-ischemic reperfusion by altering NO/redox balance, and also influence perfusion in non-ischemic conditions.

### *β*_3_AR Stimulation Is Effective in Improving Hind Limb Ischemia of Type 1 Diabetic Mice

**FIGURE 4:**
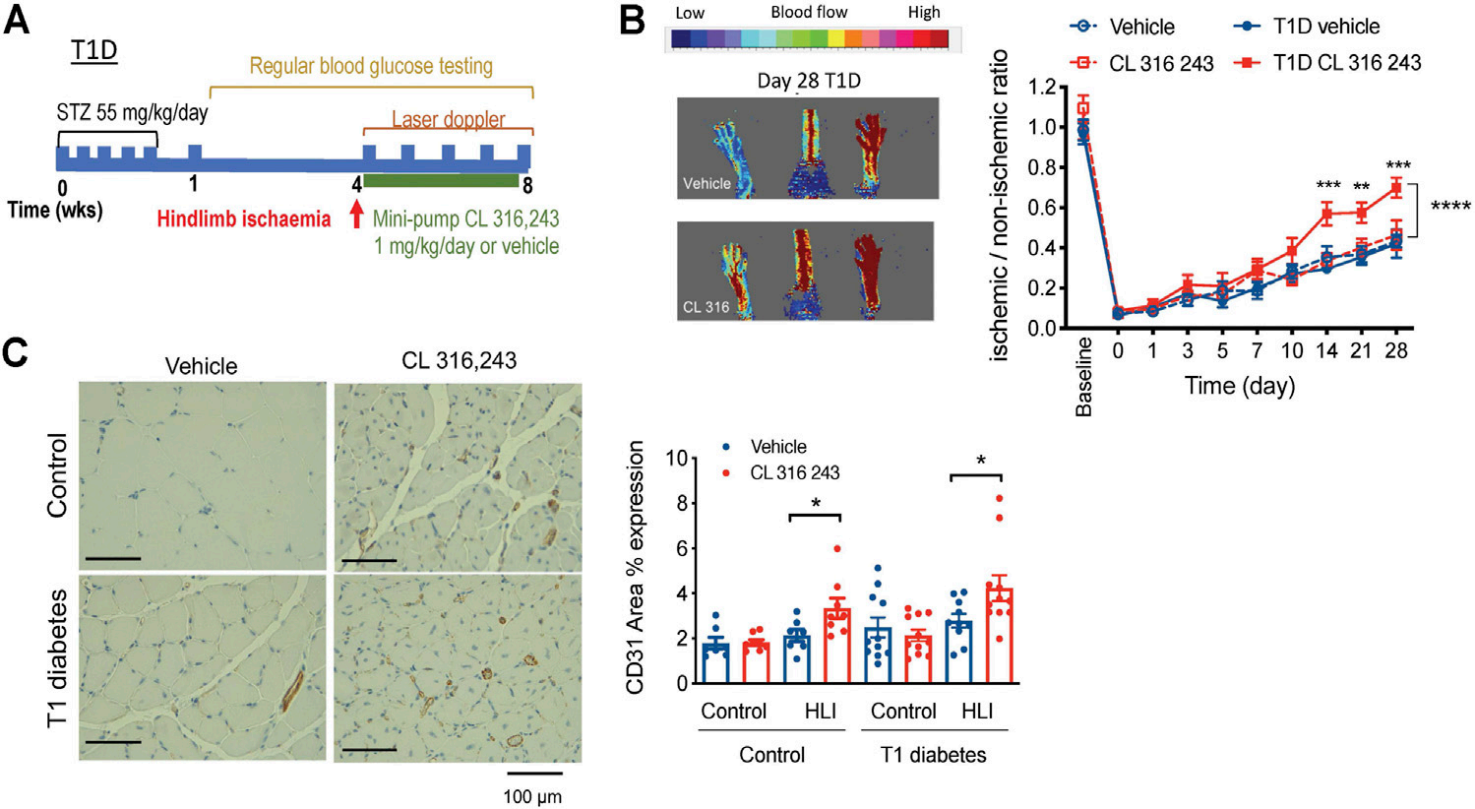
Effect of β3AR stimulation in hind limb ischemia, as measured by laser doppler imaging, in type 1 diabetic mice. **(A)** Schematic diagram showing the study protocol. **(B)** Representative image in type 1 diabetic mouse at the end of the study (day 28) and **(C)** representative image of CD31 staining (left). Right, ratio of perfusion in ischemic to non-ischemic limbs in citrate-buffer control (*n* = 7–8) and type 1 diabetes (T1D, *n* = 10) mice treated with vehicle (saline) or CL 316,243 (1 mg/kg/day, s. c. 28 days). Mean ± SEM; *p < 0.05, **p < 0.01 ***p < 0.001 vs. vehicle by 2-way ANOVA with Bonferroni post-hoc analysis. **(C)** CD31 expression in hind limbs post-ischemia in control and diabetic mice treated with vehicle or CL 316,243. Representative images show CD31 stain in brown. Mean ± SEM; *p < 0.05, **p < 0.01 vs. vehicle by 1-way ANOVA with Bonferroni post-hoc analysis; *n* = 8–10.

Diabetics have impaired angiogenesis and other vascular complications and are at increased risk of developing PAD (Thiruvoipati et al., 2015). We next wanted to examine whether *β*_3_AR stimulation could promote angiogenesis in diabetes. We first used a well-validated model of streptozotocin (STZ)-induced type 1 diabetes. Blood glucose levels were significantly elevated within a week of STZ injection in type 1 diabetes mice and remained high for the duration of the 8-weeks protocol. Hind limb ligation was conducted four weeks after the onset of type 1 diabetes, when the disease phenotype was well-established (Figure 4A; Table 1). Type 1 diabetes mice had lower body weight than their non-diabetic counterparts (Table 1). Treatment with the *β*_3_AR agonist CL 316,243 had no effect on body weight or non-fasted blood glucose levels (Table 1).

**TABLE 1:** Weight and blood glucose data for diabetic mouse study.

	Control		Diabetes		
**Type 1 diabetes model**	Vehicle (*n* = 7)	CL 316,243 (*n* = 8)	Vehicle (*n* = 11)	CL 316,243 (*n* = 11)	*p* Value
**Baseline**					
Body weight (g)	25.1 ± 0.5	25.1 ± 0.4	24.4 ± 0.5	24.6 ± 0.5	NS
Blood glucose (mmol/L)	10.8 ± 0.9	9.4 ± 0.5	8.7 ± 0.6	9.5 ± 0.5	NS
**Day 0 hind limb ischemia**					
Body weight (g)	30.8 ± 0.3	29.9 ± 0.6	26.3 ± 0.8^##^	26.3 ± 0.9^##^	0.0006
Blood glucose (mmol/L)	11.2 ± 1.0	9.7 ± 1.0	23.3 ± 2.7^##^	26.0 ± 2.4^####^	<0.0001
**Day 28 hind limb ischemia**					
Body weight (g)	31.4 ± 0.4	31.2 ± 0.5	26.9 ± 1.2^##^	27.6 ± 0.9^#^	0.002
Blood glucose (mmol/L)	10.1 ± 0.5	10.3 ± 0.6	19.8 ± 3.1	26.5 ± 2.7^###^	0.0002
**Type 2 diabetes model**	Vehicle (*n* = 12)	CL 316,243 (*n* = 11)	Vehicle (*n* = 13)	CL 316,243 (*n* = 14)	*p* Value
**Baseline**					
Body weight (g)	24.7 ± 0.5	24.9 ± 0.6	24.4 ± 0.5	24.6 ± 0.5	NS
Blood glucose (mmol/L)	10.0 ± 0.3	10.2 ± 0.5	10.7 ± 0.4	9.8 ± 0.3	NS
**Day 0 hind limb ischemia**					
Body weight (g)	34.7 ± 0.8	35.6 ± 0.7	33.6 ± 1.2	37.3 ± 2.3	NS
Blood glucose (mmol/L)	11.7 ± 0.4	11.1 ± 0.5	28.4 ± 1.7^####^	26.7 ± 1.8^####^	<0.0001
**Day 28 hind limb ischemia**					
Body weight (g)	34.3 ± 0.7	33.1 ± 0.5	32.7 ± 1.0	35.5 ± 1.8	NS
Blood glucose (mmol/L)	12.5 ± 0.4	11.6 ± 0.5	25.7 ± 2.2^####^	23.5 ± 2.2^####^	0.0002

Statistical analysis by 1-way ANOVA. ^#^ *p* < 0.05, ^##^ *p* < 0.01, ^###^ *p* < 0.001, ^####^ *p* < 0.0001 vs. Citrate control. NS, not significant. No differences detected between Vehicle and CL 316,243-treated mice.

Following hindlimb ischemia, *β*_3_AR stimulation resulted in accelerated reperfusion in type 1 diabetes mice, as shown by ∼20 greater ischemic-non-ischemic ratio from 14 days onwards (Figure 4B). The citrate-buffer treated mice mirrored the results of non-diabetic mice, where perfusion ratio of CL 316,243-treated mice was not different from vehicle controls, and this was also the case from 14 to 28 days post-ischemia (Figure 4B). We next assessed vascularization and showed greater CD31 ^+^ staining in ischemic hindlimbs of mice treated with CL 316,243, in both the type 1 diabetes and the non-diabetic mice (Figure 4D).

### *β*_3_AR Stimulation Ameliorates Dysregulated Redox Signaling After Hind Limb Ischemia

**FIGURE 5:**
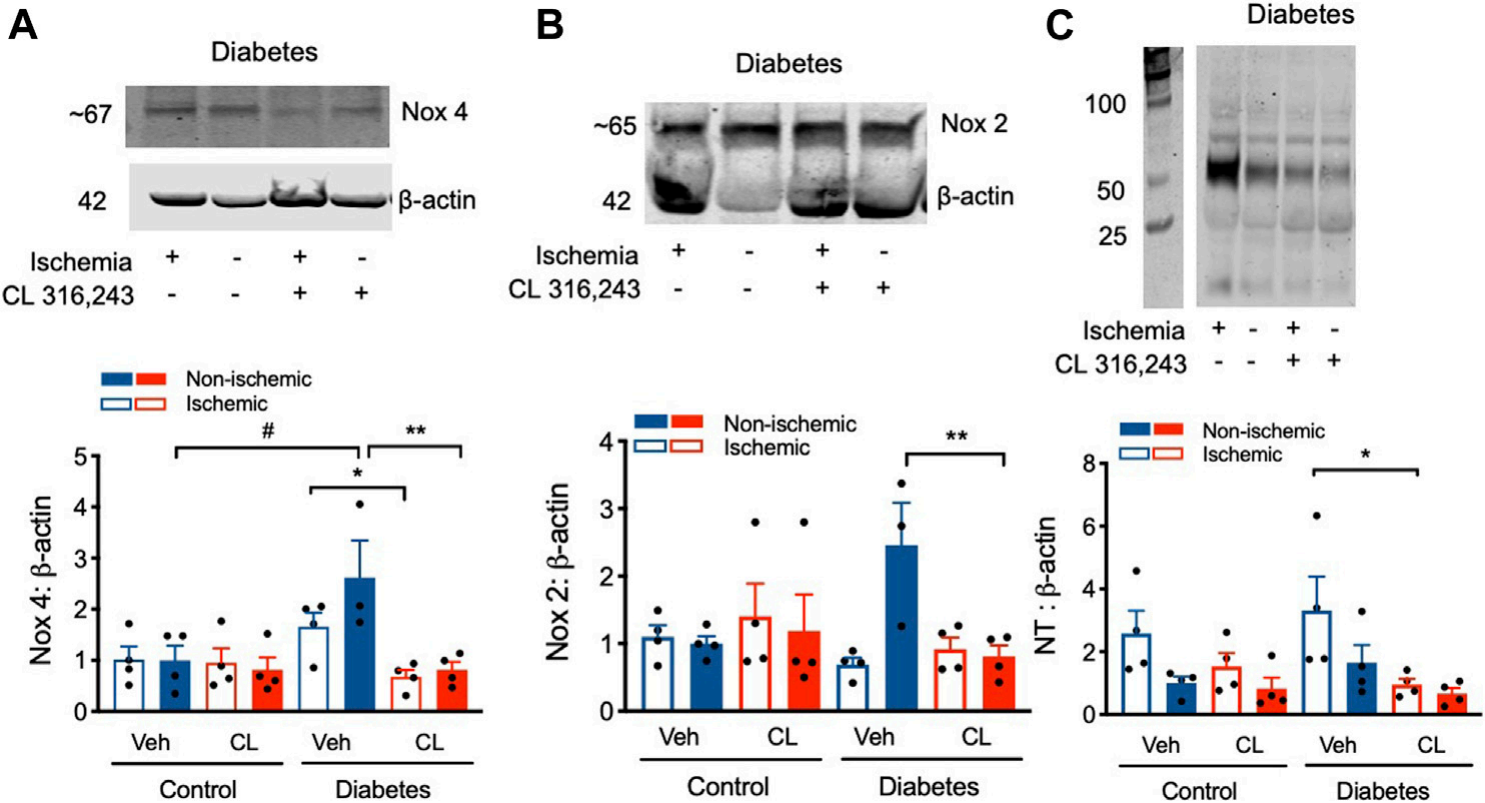
Modified redox signaling after hind limb ischemia was normalized by β_3_AR stimulation. **(A)** Upper panel, representative blot of Nox4 protein expression in hind limb tissue from diabetic mice. Lower panel, quantification. **(B)** Upper panel, representative blot of Nox2 protein expression in hind limb tissue from diabetic mice. Lower panel, quantification. **(C)** Upper panel, representative blot of nitrotyrosine expression in hind limb tissue from diabetic mice; Lower panel, quantification of nitrotyrosine expression; In all groups protein expression is shown relative to citrate vehicle non-ischemic limb. Data presented as mean ± SEM. Statistical analysis by 1-way ANOVA with Bonferroni post-hoc analysis. ^#^ *p* < 0.05 vs. diabetes vs. citrate; *p < 0.05, **p < 0.01 CL 316,243 vs. vehicle; *n* = 4.

*β*_3_AR stimulation can modulate redox-NO balance (Karimi Galougahi et al., 2016). We therefore examined multiple readouts important in regulating this pathway including assessment of NOX expression and levels of nitrotyrosine, a surrogate marker of reactive nitrogen species such as peroxynitrite. Compared to control, Nox 4 expression was elevated ∼2-3 fold in diabetes, in both the ischemic and non-ischemic limb (Figure 5A). However, this diabetes-induced elevation in Nox4 expression was markedly reduced with CL treatment, back to control levels. Similar findings were observed for Nox 2 protein expression, but changes were only observed in the non-ischemic limb (Figure 5B). Consistent with changes to the redox state in diabetes, nitrotyrosine protein levels were increased 4-fold in ischemic hind limbs of type 1 diabetes mice relative to non-ischemic limbs in control mice. *β*_3_AR agonist treatment profoundly protected against ischemia-induced nitrotyrosylation, decreasing levels by >70% in the diabetic mice (Figure 5C). These findings indicate that *β*_3_AR agonist stimulation normalizes the dysregulated redox balance in diabetes.

### *β*_3_AR Stimulation Abrogates Endothelial NO Synthase Glutathionylation in Ischemic Limbs of Diabetic Mice

**FIGURE 6:**
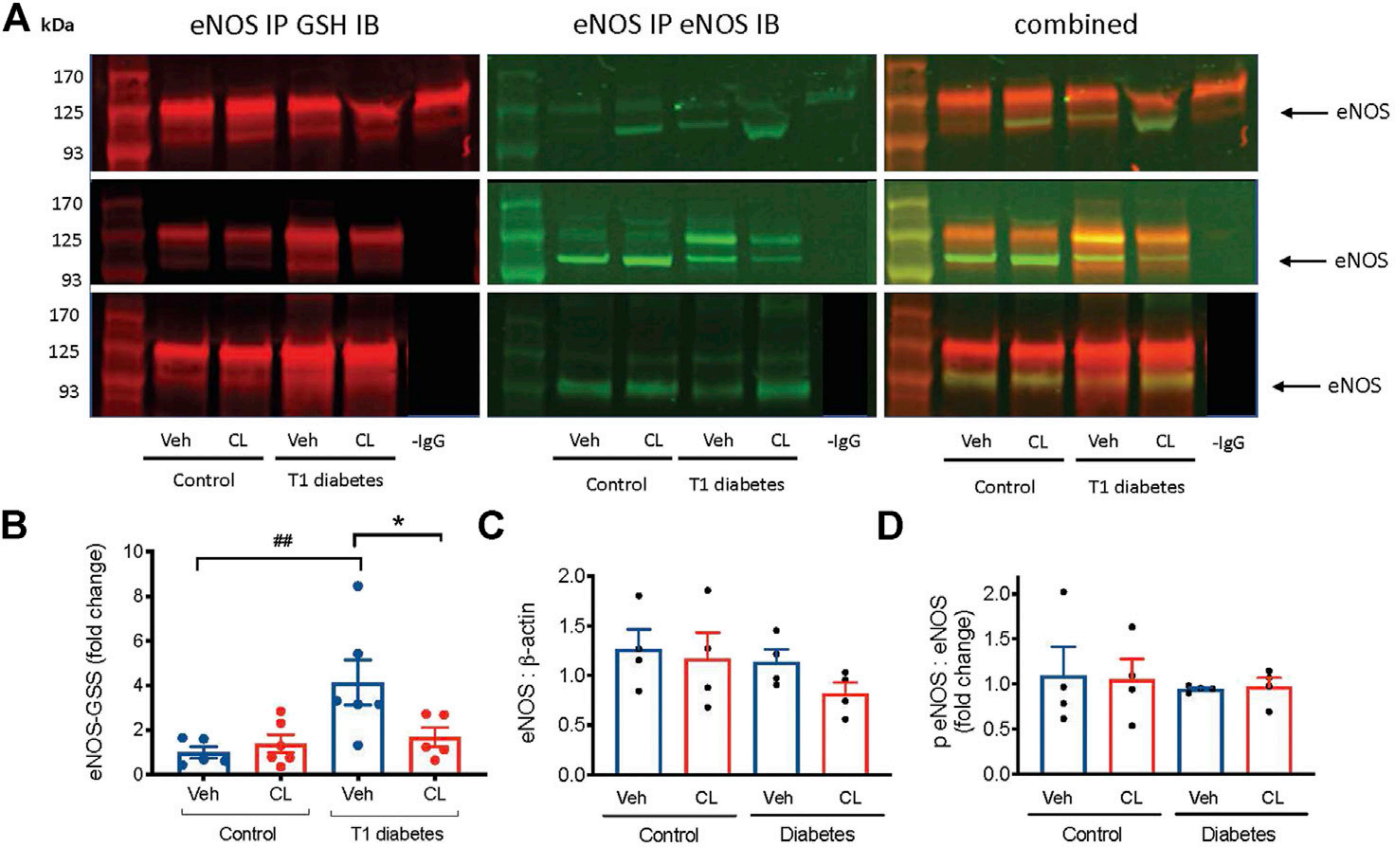
Glutathionylation of eNOS (eNOS-GSS) in ischemic hind limb samples from type 1 (T1) diabetic mice. **(A)** Representative images of immunoblots (IB) performed on protein fractions following eNOS immunoprecipitation (IP). On the left the expression of GSH, detected at 680 nm is shown and in the middle, the simultaneous expression of eNOS, detected at 800 nm are shown. The right panel shows the merged image of both detection channels. The negative control, -IgG antibody used during IP, is shown only in the top panel. All samples were extracted and run simultaneously. **(B)** Summary data of eNOS glutathionylation shown as the ratio of glutathionylated eNOS to total eNOS in ischemic hind limb samples. **(C)** eNOS relative to *β*-actin expression in total ischemic hindlimb lysate from immunoblot. **(D)** phosphorylated eNOS (serine 1177) relative to total eNOS in the ischemic hindlimb. Summary data presented as mean ± SEM. Statistical analysis by 1-way ANOVA with Bonferroni post-hoc analysis. ^##^ *p* < 0.01 vs. citrate, *p < 0.05 vs. vehicle.

A key mechanism of eNOS uncoupling is post-translational modification involving glutathione adduct cysteine residues on the reductase domain of eNOS (Chen et al., 2010). Biochemical studies performed to quantify the effect of eNOS uncoupling by this mechanism show a decrease in NO production by ∼70%, and an increase in superoxide by 5-fold (Chen et al., 2010). To investigate the possible role of eNOS glutathionylation in the ischemic limbs, and the benefits of CL 316,243, we performed eNOS immunoprecipitation and detected the oxidized glutathione and eNOS co-expression. Glutathionylation of eNOS was increased >3 fold in the ischemic limbs of type 1 diabetes mice and this was largely abolished in mice treated with the *β*_3_AR agonist (Figure 6A). eNOS expression was unaltered (Figure 6B). Phosphorylation of eNOS at serine 1,177, which is sensitive to oxidative stress and can result in eNOS uncoupling, did not appear to be affected by either type 1 diabetes or *β*_3_AR stimulation (Figure 6D).

### *β*_3_AR Stimulation Also Promotes Reperfusion in a High-Fat Fed Diabetic Model

**FIGURE 7:**
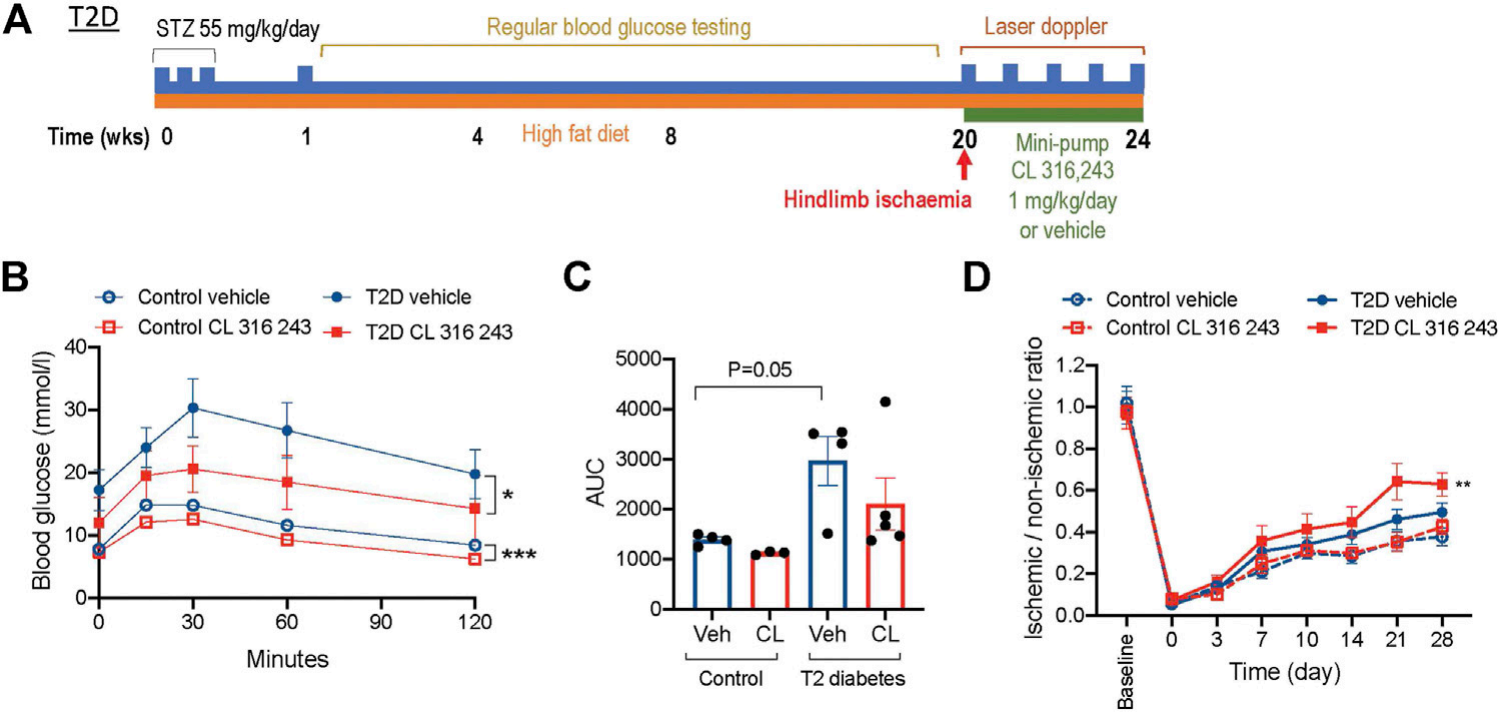
β_3_AR stimulation improves glucose tolerance and recovery from post-ischemic injury in type 2 diabetes. **(A)** Schematic of the type 2 diabetes (T2D) protocol. **(B)** Glucose tolerance tests in a cohort of citrate-buffer control (*n* = 3–4) and T2D mice treated with vehicle or CL 316,243 (*n* = 4–5). **(C)** Area under the curve (AUC) analysis of glucose tolerance results. **(D)** Ischemic to non-ischemic ratio of perfusion in citrate-buffer treated control (*n* = 12) and T2D (*n* = 13) mice measured by laser doppler imaging. Data presented as mean ± SEM; *p < 0.05, **p < 0.01, ***p < 0.001 vs. vehicle by 2-way ANOVA.

While we have shown a potential therapeutic role for CL 316,243 in type 1 diabetes, it is also highly relevant for the PAD population to investigate the effect in a model that recapitulates features of type 2 diabetes. We utilized a well-validated and characterized model of insulin resistance and type 2 diabetes (Gilbert et al., 2011). Mice were fed a high-fat diet for 20 weeks after instigation of low-dose STZ (Figure 7A). This resulted in a hyperglycemic model that were protected from the metabolic disturbance causing substantial weight-loss seen in the type 1 diabetes model (Table 1). The body weights were similar in citrate-buffer and type 2 diabetes mice prior to hind limb ischemia, and not affected by CL 316,243 infusion after the ligation surgery (Table 1). Blood glucose levels rose rapidly and were consistently in the hyperglycemic range for the duration of the protocol. Prior to and after CL 316,243 infusion, non-fasted blood glucose levels were similar in both type 2 diabetes groups. Interestingly, when mice were fasted for glucose tolerance tests, blood glucose levels appeared lower in the CL 316,243 group, although this did not reach significant difference. Glucose tolerance was improved in type 2 diabetic mice treated with CL 316,243 (Figure 7B). Glucose tolerance was even improved in CL 316,243 treated non-hyperglycemic, non-diabetic controls (Figure 7C). Importantly, the protective effects of *β*_3_AR stimulation on diabetic ischemic injury were again evident, with augmented reperfusion post-ischemia in type 2 diabetes mice treated with CL 316,243 (Figure 7D).

## Discussion

There is a clear unmet need for medical treatment options targeting underlying PAD mechanisms driving both atherosclerosis as well as tissue ischemia to improve quality of life for PAD patients and reduce morbidity and mortality. Whilst surgical and percutaneous approaches to revascularization have been partially successful, this is expensive and not without risk to the patient, including the need for recurrent procedures. Therapeutic angiogenesis and improvements of microvascular function are a promising strategy. Here we demonstrate for the first time that *β*_3_AR stimulation improves eNOS activity and redox balance in a pre-clinical model of PAD and this translates to significant improvement in limb perfusion in mice with vascular complications of diabetes. In addition to restoration of NO/redox balance CL 316,243 stimulated growth of new blood vessels. There may also have been contribution of vasodilation as there was evidence of systemic improvements in perfusion not specific to the ischemic limb. Whilst many preclinical studies have been challenging to translate to humans, the safe and well accepted use of the *β*_3_AR agonist, Mirabegron, for patients with overactive bladder syndrome makes the opportunity for drug repurposing and translation of our findings immediately feasible. Thus, our findings have direct relevance for the >200 million people worldwide suffering from atherosclerotic PAD, particularly those with the co-morbidity of diabetes.

Our findings provide clear evidence that *β*_3_AR stimulation can promote angiogenesis *in vitro*, in cultured microvascular, umbilical vein and ECFCs, consistent with previous reports from studies using retinal endothelial cells (Schena and Caplan, 2019). We demonstrated that the pro-angiogenic effects of the *β*_3_AR agonist are due, at least in part, to improved NO bioavailability. This is consistent with previous findings showing that *β*_3_AR-mediated vascular relaxation is mediated by NO signaling (Dessy et al., 2005; Karimi Galougahi et al., 2016). The activation of eNOS, including by VEGF, often involves phosphorylation of serine 1,177 by Akt (Dimmeler et al., 1999; Karar and Maity, 2011). However, whilst we observed β3AR-induced increases in eNOS activity in both ischemic and non-ischemic limbs, this was not associated with an increase in phosphorylation.

Our findings of *β*_3_AR stimulation promoting angiogenesis are well supported by previous studies (Schena and Caplan, 2019), however we are the first to demonstrate the functional outcome in a model of PAD. Our demonstration of the pro-angiogenesis capacity of the *β*_3_AR agonist in relevant ECFCs from patients with cardiovascular disease provides proof-of-concept that *β*_3_AR stimulation may be effective in patient populations. Our somewhat surprising findings revealed that significant angiogenesis in response to CL 316,243 did not occur in the cells from relatively healthy participants. This may indicate that *β*_3_AR stimulation is more effective in a pathological state and is supported by our animal studies showing a stronger role for *β*_3_AR stimulation in diabetic compared to healthy mice. Whilst this may be due to numerous modifications in inflammatory and oxidative signaling under disease conditions, it is likely to be at least partially dependent on the restoration of low NO bioavailability and redistribution or upregulation of *β*_3_ARs (Michel et al., 2020; Schobesberger et al., 2020).

The “uncoupling” of eNOS refers to not only a decrease in NO production by the enzyme, but also the increased generation of superoxide. This occurs by a number of different mechanisms, including oxidation of the cofactor BH_4_ (Landmesser et al., 2003; Crabtree et al., 2009), depletion of substrate l-arginine (Zweier et al., 2011) and NADPH oxidase-induced superoxide production (Förstermann and Li, 2011) or post-translational modification by glutathionylation of cysteines 689 and 908 on the eNOS reductase domain (eNOS-GSS) (Chen et al., 2010). *β*_3_AR stimulation resulted in reciprocal decreases in superoxide and increased eNOS activity and the improved eNOS-GSS status, at least in the diabetic mice. This is supportive of decreased glutathionylated or “uncoupled” eNOS being a significant mediator contributing to improved limb perfusion. The contribution of eNOS-GSS to endothelial superoxide production under physiological and pathophysiological stimuli has been previously highlighted by our group where we demonstrated almost complete loss of angiotensin II-induced superoxide production in endothelial cells expressing a “redox-null” eNOS lacking the reactive cysteine required for glutathionylation (Galougahi et al., 2014). When we investigated the effect of *β*_3_AR agonism on the source of superoxide we found that whilst there was no effect on Nox2, Nox4 expression was significantly reduced in the diabetic model. This is suggestive that Nox4 may be contributing, at least partially, to the dysregulated redox signaling in the hindlimb, and that the decreased expression of Nox 4 may be mediating some of the benefits of CL 316,243. Nox4 does have a central role in the renal redox dysregulation in diabetic models (Thallas-Bonke et al., 2014; Jha et al., 2016). However, our findings are inconsistent with the previously demonstrated role for the Nox 4 isoform in hind limb ischemia (Steven et al., 2017), with Nox4-derived H_2_O_2_ actually promoting angiogenesis. Interestingly, it was recently shown that β3AR stimulation can reduce LPS-induced NADPH oxidase activity and Nox 2 expression in macrophages (Hadi et al., 2017). Therefore, it is clear that further work should focus on the complex interactions of Nox isoforms with β3ARs as a promising strategy to lower pathological superoxide generation.

Whilst S-glutathionylation is well recognized to be protective against irreversible oxidation in some settings (Dalle-Donne et al., 2009) (eg., SERCA (Adachi et al., 2004; Ying et al., 2007)), it has also been clearly demonstrated to acutely inhibit the function of proteins, depending on the protein and signaling pathway involved, and the steric effects in this context (Takata et al., 2020). Our finding that decreased S-glutathionylation of eNOS is associated with improved eNOS function and limb perfusion fits with the biochemical evidence that this chemical modification is causally responsible for a 70% reduction in NO and 5-fold increase in superoxide production (Chen et al., 2010).

A continuing challenge for discovery of new therapeutics for PAD is the relatively poor translation to clinical practice of candidates so far (Krishna et al., 2016). Mirabegron, is approved for use in multiple countries, is well tolerated and has a proven safety record (Warren et al., 2016), so poses an excellent opportunity to proceed directly to clinical trial without the need for further use of animals models. This drug could be readily repurposed for use by PAD patients if it is proven to be effective in clinical trial. We have begun recruitment for the STimulating *β*_3_ Adrenergic Receptors to improve clinical outcomes in patients with Peripheral arterial disease (STAR-PAD) trial which is a phase II, double-blinded, randomized controlled trial (Australian New Zealand Clinical Trials Registry: 12619000,423,112) powered to determine the potential benefit of Mirabegron on the primary endpoint of peak walk time on a standardized treadmill protocol. Additional secondary endpoints will allow potential mechanisms to be explored. There are a few potential limitations of a *β*_3_AR stimulation strategy. Firstly, an important consideration for a β3AR stimulation strategy in patients with peripheral arterial disease is the contraindication in patients with extreme hypertension (systolic blood pressure >180 mm Hg), due to a possible potentiating effect. However, the risk for patients with systolic blood pressure lower than 180 mm Hg is thought to be minimal. Meta-analysis of the largest randomized control trials has shown that hypertension accounts for ∼8.7% of treatment-emergent adverse events due to mirabegron compared with 8.5% placebo and that compared to placebo, mirabegron causes an increase in blood pressure of only ∼1 mmHg (Rosa et al., 2016). This is unlikely to have any opposing negative effects on peripheral perfusion. Secondly, as with all promotors of angiogenesis, could be a propensity for pro-tumorigenic effects. It has been suggested that caution should be taken when prescribing Mirabegron due to potential for causing cancers (Goldstein & Mascitelli, 2018) with *β*_3_ARs have been shown to be overexpressed in some tumors (Rains et al., 2017). However, a cohort study of 119,912 adults in the United Kingdom concluded that Mirabegron use was not causally linked to cancer incidence (Kaye et al., 2017).

## Conclusion

In summary, we have demonstrated a clear benefit of using a selective *β*_3_AR agonist in a pre-clinical model of PAD. The improved reperfusion was largely attributed to increased eNOS activity. We confirmed the direct role of CL 316,243 in stimulating angiogenesis using well characterized *in vitro* assays with human endothelial cells as a proof of concept. Given the wide availability of a safe and well tolerated *β*_3_AR agonist, the striking findings of this study have led to the initiation of a phase 2 clinical trial. If successful, this treatment could be rapidly implemented into clinical practice for a common, debilitating disease state with a paucity of therapeutic options.

## Data Availability

The original contributions presented in the study are included in the article/Supplementary Material, further inquiries can be directed to the corresponding authors.

## References

[B1] Adachi T. Weisbrod R. M. Pimentel D. R. Ying J. Sharov V. S. Schöneich C. (2004). S-glutathiolation by Peroxynitrite Activates SERCA during Arterial Relaxation by Nitric Oxide. Nat. Med. 10, 1200–1207. 10.1038/nm1119 15489859

[B2] Balligand J.-L. (2016). Cardiac Salvage by Tweaking with Beta-3-Adrenergic Receptors. Cardiovasc. Res. 111, 128–133. 10.1093/cvr/cvw056 27001422

[B3] Berlan M. Galitzky J. Bousquet-Melou A. Lafontan M. Montastruc J. L. (1994). Beta-3 Adrenoceptor-Mediated Increase in Cutaneous Blood Flow in the Dog. J. Pharmacol. Exp. Ther. 268, 1444–1451. 7908057

[B4] Brown D. I. Griendling K. K. (2015). Regulation of Signal Transduction by Reactive Oxygen Species in the Cardiovascular System. Circ. Res. 116, 531–549. 10.1161/circresaha.116.303584 25634975 PMC4392388

[B5] Bubb K. J. Ritchie R. H. Figtree G. A. (2018). Modified Redox Signaling in Vasculature after Chronic Infusion of the Insulin Receptor Antagonist, S961. Microcirculation 26, e12501. 10.1111/micc.12501 30178465

[B6] Bundgaard H. Liu C.-C. Garcia A. Hamilton E. J. Huang Y. Chia K. K. M. (2010). β 3 Adrenergic Stimulation of the Cardiac Na + -K + Pump by Reversal of an Inhibitory Oxidative Modification. Circulation 122, 2699–2708. 10.1161/circulationaha.110.964619 21135361

[B7] Cannavo A. Koch W. J. (2017). Targeting β3-Adrenergic Receptors in the Heart: Selective Agonism and β-Blockade. J. Cardiovasc. Pharmacol. 69, 71–78. 10.1097/fjc.0000000000000444 28170359 PMC5295490

[B8] Chen C.-A. Wang T.-Y. Varadharaj S. Reyes L. A. Hemann C. Talukder M. A. H. (2010). S-glutathionylation Uncouples eNOS and Regulates its Cellular and Vascular Function. Nature 468, 1115–1118. 10.1038/nature09599 21179168 PMC3370391

[B9] Clayton Z. E. Yuen G. S. C. Sadeghipour S. Hywood J. D. Wong J. W. T. Huang N. F. (2017). A Comparison of the Pro-angiogenic Potential of Human Induced Pluripotent Stem Cell Derived Endothelial Cells and Induced Endothelial Cells in a Murine Model of Peripheral Arterial Disease. Int. J. Cardiol. 234, 81–89. 10.1016/j.ijcard.2017.01.125 28209385

[B10] Crabtree M. J. Tatham A. L. Al-Wakeel Y. Warrick N. Hale A. B. Cai S. (2009). Quantitative Regulation of Intracellular Endothelial Nitric-Oxide Synthase (eNOS) Coupling by Both Tetrahydrobiopterin-eNOS Stoichiometry and Biopterin Redox Status. J. Biol. Chem. 284, 1136–1144. 10.1074/jbc.m805403200 19011239

[B11] Dalle-Donne I. Rossi R. Colombo G. Giustarini D. Milzani A. (2009). Protein S-Glutathionylation: a Regulatory Device from Bacteria to Humans. Trends Biochem. Sci. 34, 85–96. 10.1016/j.tibs.2008.11.002 19135374

[B12] Dessy C. Saliez J. Ghisdal P. Daneau G. Lobysheva I. Frérart F. (2005). Endothelial β 3 -Adrenoreceptors Mediate Nitric Oxide-dependent Vasorelaxation of Coronary Microvessels in Response to the Third-Generation β-Blocker Nebivolol. Circulation 112, 1198–1205. 10.1161/circulationaha.104.532960 16116070

[B13] Dimmeler S. Fleming I. Fisslthaler B. Hermann C. Busse R. Zeiher A. M. (1999). Activation of Nitric Oxide Synthase in Endothelial Cells by Akt-dependent Phosphorylation. Nature 399, 601–605. 10.1038/21224 10376603

[B14] European Stroke O. Tendera M. Aboyans V. Bartelink M. L. Baumgartner I. Clément D. (2011). ESC Guidelines on the Diagnosis and Treatment of Peripheral Artery Diseases: Document Covering Atherosclerotic Disease of Extracranial Carotid and Vertebral, Mesenteric, Renal, Upper and Lower Extremity Arteries: the Task Force on the Diagnosis and Treatment of Peripheral Artery Diseases of the European Society of Cardiology (ESC). Eur. Heart J. 32, 2851–2906. 10.1093/eurheartj/ehr211 21873417

[B15] Förstermann U. Li H. (2011). Therapeutic Effect of Enhancing Endothelial Nitric Oxide Synthase (eNOS) Expression and Preventing eNOS Uncoupling. Br. J. Pharmacol. 164, 213–223. 10.1111/j.1476-5381.2010.01196.x 21198553 PMC3174401

[B17] Galougahi K. K. Liu C. C. Gentile C. Kok C. Nunez A. Garcia A. (2014). Glutathionylation Mediates Angiotensin II-Induced eNOS Uncoupling, Amplifying NADPH Oxidase-dependent Endothelial Dysfunction. J. Am. Heart Assoc. 3, e000731. 10.1161/jaha.113.000731 24755153 PMC4187489

[B16] Galougahi K. K. Liu C.-C. Bundgaard H. Rasmussen H. H. (2012). β-Adrenergic Regulation of the Cardiac Na+-K+ ATPase Mediated by Oxidative Signaling. Trends Cardiovasc. Med. 22, 83–87. 10.1016/j.tcm.2012.06.017 23040838

[B18] Gauthier C. Langin D. Balligand J.-L. (2000). β3-Adrenoceptors in the Cardiovascular System. Trends Pharmacol. Sci. 21, 426–431. 10.1016/s0165-6147(00)01562-5 11121573

[B19] Gerhard-Herman M. Gardin J. M. Jaff M. Mohler E. Roman M. Naqvi T. Z. (2006). Guidelines for Noninvasive Vascular Laboratory Testing: a Report from the American Society of Echocardiography and the Society of Vascular Medicine and Biology. J. Am. Soc. Echocardiography 19, 955–972. 10.1016/j.echo.2006.04.019 16880090

[B20] Gilbert E. R. Fu Z. Liu D. (2011). Development of a Nongenetic Mouse Model of Type 2 Diabetes. Exp. Diabetes Res. 2011, 416254. 10.1155/2011/416254 22164157 PMC3226533

[B21] Goldstein M. R. Mascitelli L. (2018). Innervation of the Tumor Microenvironment-Letter. Cancer Res. 78, 6022. 10.1158/0008-5472.CAN-18-2198 30275020

[B22] Hadi T. Douhard R. Dias A. M. M. Wendremaire M. Pezzè M. Bardou M. (2017). Beta3 Adrenergic Receptor Stimulation in Human Macrophages Inhibits NADPHoxidase Activity and Induces Catalase Expression via PPARγ Activation. Biochim. Biophys. Acta (Bba) - Mol. Cel Res. 1864, 1769–1784. 10.1016/j.bbamcr.2017.07.003 28723418

[B23] Hamburg N. M. Creager M. A. (2017). Pathophysiology of Intermittent Claudication in Peripheral Artery Disease. Circ. J. 81, 281–289. 10.1253/circj.cj-16-1286 28123169

[B24] Iyer S. R. Annex B. H. (2017). Therapeutic Angiogenesis for Peripheral Artery Disease. JACC: Basic Translational Sci. 2, 503–512. 10.1016/j.jacbts.2017.07.012 PMC5802410 29430558

[B25] Jha J. C. Thallas-Bonke V. Banal C. Gray S. P. Chow B. S. M. Ramm G. (2016). Podocyte-specific Nox4 Deletion Affords Renoprotection in a Mouse Model of Diabetic Nephropathy. Diabetologia 59, 379–389. 10.1007/s00125-015-3796-0 26508318 PMC6450410

[B26] Karar J. Maity A. (2011). PI3K/AKT/mTOR Pathway in Angiogenesis. Front. Mol. Neurosci. 4, 51. 10.3389/fnmol.2011.00051 22144946 PMC3228996

[B27] Karimi Galougahi K. Antoniades C. Nicholls S. J. Channon K. M. Figtree G. A. (2015). Redox Biomarkers in Cardiovascular Medicine. Eur. Heart J. 36, 1576. 10.1093/eurheartj/ehv126 25888005

[B28] Karimi Galougahi K. Liu C. C. Garcia A. Gentile C. Fry N. A. Hamilton E. J. (2016). beta3 Adrenergic Stimulation Restores Nitric Oxide/Redox Balance and Enhances Endothelial Function in Hyperglycemia. J. Am. Heart Assoc. 5. 10.1161/jaha.115.002824 PMC4802476 26896479

[B29] Kaye J. A. Margulis A. V. Fortuny J. McQuay L. J. Plana E. Bartsch J. L. (2017). *Cancer* Incidence after Initiation of Antimuscarinic Medications for Overactive Bladder in the United Kingdom: Evidence for Protopathic Bias. Pharmacotherapy 37, 673–683. 10.1002/phar.1932 28370075 PMC5518180

[B30] Kenjale A. A. Ham K. L. Stabler T. Robbins J. L. Johnson J. L. Vanbruggen M. (1985). Dietary Nitrate Supplementation Enhances Exercise Performance in Peripheral Arterial Disease. J. Appl. Physiol. 110, 1582–1591. 10.1152/japplphysiol.00071.2011 PMC3119136 21454745

[B31] Kott K. A. Vernon S. T. Hansen T. Yu C. Bubb K. J. Coffey S. (2019). Biobanking for Discovery of Novel Cardiovascular Biomarkers Using Imaging-Quantified Disease Burden: Protocol for the Longitudinal, Prospective, BioHEART-CT Cohort Study. BMJ Open 9, e028649. 10.1136/bmjopen-2018-028649 PMC6756427 31537558

[B33] Krishna S. M. Omer S. M. Li J. Morton S. K. Jose R. J. Golledge J. (2020). Development of a Two-Stage Limb Ischemia Model to Better Simulate Human Peripheral Artery Disease. Sci. Rep. 10, 3449. 10.1038/s41598-020-60352-4 32103073 PMC7044206

[B32] Krishna S. M. Omer S. M. Golledge J. (2016). Evaluation of the Clinical Relevance and Limitations of Current Pre-clinical Models of Peripheral Artery Disease. Clin. Sci. (Lond). 130, 127–150. 10.1042/cs20150435 26678170

[B34] Landmesser U. Dikalov S. Price S. R. McCann L. Fukai T. Holland S. M. (2003). Oxidation of Tetrahydrobiopterin Leads to Uncoupling of Endothelial Cell Nitric Oxide Synthase in Hypertension. J. Clin. Invest. 111, 1201–1209. 10.1172/jci200314172 12697739 PMC152929

[B35] Lerman A. Zeiher A. M. (2005). Endothelial Function. Circulation 111, 363–368. 10.1161/01.cir.0000153339.27064.14 15668353

[B36] Mahoney E. M. Wang K. Cohen D. J. Hirsch A. T. Alberts M. J. Eagle K. (2008). One-year Costs in Patients with a History of or at Risk for Atherothrombosis in the United States. Circ. Cardiovasc. Qual. Outcomes 1, 38–45. 10.1161/circoutcomes.108.775247 20031786

[B37] Michel L. Y. M. Farah C. Balligand J. L. (2020). The Beta3 Adrenergic Receptor in Healthy and Pathological Cardiovascular Tissues. Cells 9. 10.3390/cells9122584 PMC7761574 33276630

[B38] Norgren L. Hiatt W. R. Dormandy J. A. Nehler M. R. Harris K. A. Fowkes F. G. (2007). Inter-Society Consensus for the Management of Peripheral Arterial Disease (TASC II). J. Vasc. Surg. 45, S5–S67. 10.1016/j.jvs.2006.12.037 17223489

[B39] Prakoso D. De Blasio M. J. Qin C. Rosli S. Kiriazis H. Qian H. (2017). Phosphoinositide 3-kinase (P110α) Gene Delivery Limits Diabetes-Induced Cardiac NADPH Oxidase and Cardiomyopathy in a Mouse Model with Established Diastolic Dysfunction. Clin. Sci. (Lond). 131, 1345–1360. 10.1042/cs20170063 28487469

[B40] Rains S. L. Amaya C. N. Bryan B. A. (2017). Beta-adrenergic Receptors Are Expressed across Diverse Cancers. Oncoscience 4, 95–105. 10.18632/oncoscience.357 28966942 PMC5616202

[B41] Riedhammer C. Halbritter D. Weissert R. (2016). Peripheral Blood Mononuclear Cells: Isolation, Freezing, Thawing, and Culture. Methods Mol. Biol. 1304, 53–61. 10.1007/7651_2014_99 25092056

[B42] Rosa G. M. Ferrero S. Nitti V. W. Wagg A. Saleem T. Chapple C. R. (2016). Cardiovascular Safety of β3-adrenoceptor Agonists for the Treatment of Patients with Overactive Bladder Syndrome. Eur. Urol. 69, 311–323. 10.1016/j.eururo.2015.09.007 26422675

[B43] Salhiyyah K. Forster R. Senanayake E. Abdel-Hadi M. Booth A. Michaels J. A. (2015). Pentoxifylline for Intermittent Claudication. Cochrane Database Syst. Rev. 9, CD005262. 10.1002/14651858.CD005262.pub3 26417854 PMC6513423

[B44] Schena G. Caplan M. J. (2019). Everything You Always Wanted to Know about Beta3-AR * (* but Were Afraid to Ask). Cells 8. 10.3390/cells8040357 PMC6523418 30995798

[B45] Schobesberger S. Wright P. T. Poulet C. Sanchez Alonso Mardones J. L. Mansfield C. Friebe A. (2020). beta3-Adrenoceptor Redistribution Impairs NO/cGMP/PDE2 Signalling in Failing Cardiomyocytes. Elife 9, e52221. 10.7554/elife.52221 32228862 PMC7138611

[B47] Shen Y. T. Cervoni P. Claus T. Vatner S. F. (1996). Differences in Beta 3-adrenergic Receptor Cardiovascular Regulation in Conscious Primates, Rats and Dogs. J. Pharmacol. Exp. Ther. 278, 1435–1443. 8819531

[B46] Shen Y. T. Zhang H. Vatner S. F. (1994). Peripheral Vascular Effects of Beta-3 Adrenergic Receptor Stimulation in Conscious Dogs. J. Pharmacol. Exp. Ther. 268, 466–473. 7905532

[B48] Steinle J. J. Booz G. W. Meininger C. J. Day J. N. E. Granger H. J. (2003). β3-Adrenergic Receptors Regulate Retinal Endothelial Cell Migration and Proliferation. J. Biol. Chem. 278, 20681–20686. 10.1074/jbc.m300368200 12670949

[B49] Steven S. Daiber A. Dopheide J. F. Münzel T. Espinola-Klein C. (2017). Peripheral Artery Disease, Redox Signaling, Oxidative Stress - Basic and Clinical Aspects. Redox Biol. 12, 787–797. 10.1016/j.redox.2017.04.017 28437655 PMC5403804

[B50] Takata T. Araki S. Tsuchiya Y. Watanabe Y. (2020). Oxidative Stress Orchestrates MAPK and Nitric-Oxide Synthase Signal. Int. J. Mol. Sci. 21, 21. 10.3390/ijms21228750 PMC7699490 33228180

[B51] Tate M. Prakoso D. Willis A. M. Peng C. Deo M. Qin C. X. (2019). Characterising an Alternative Murine Model of Diabetic Cardiomyopathy. Front. Physiol. 10, 1395. 10.3389/fphys.2019.01395 31798462 PMC6868003

[B52] Thallas-Bonke V. Jha J. C. Gray S. P. Barit D. Haller H. Schmidt H. H. (2014). Nox-4 Deletion Reduces Oxidative Stress and Injury by PKC-Alpha-Associated Mechanisms in Diabetic Nephropathy. Physiol. Rep. 2. 10.14814/phy2.12192 PMC4255803 25367693

[B53] Thiruvoipati T. Kielhorn C. E. Armstrong E. J. (2015). Peripheral Artery Disease in Patients with Diabetes: Epidemiology, Mechanisms, and Outcomes. Wjd 6, 961–969. 10.4239/wjd.v6.i7.961 26185603 PMC4499529

[B54] Uccioli L. Meloni M. Izzo V. Giurato L. Merolla S. Gandini R. (2018). Critical Limb Ischemia: Current Challenges and Future Prospects. Vhrm 14, 63–74. 10.2147/vhrm.s125065 PMC5927064 29731636

[B55] Warren K. Burden H. Abrams P. (2016). Mirabegron in Overactive Bladder Patients: Efficacy Review and Update on Drug Safety. Ther. Adv. Drug Saf. 7, 204–216. 10.1177/2042098616659412 27695622 PMC5014049

[B56] Ying J. Tong X. Pimentel D. R. Weisbrod R. M. Trucillo M. P. Adachi T. (2007). Cysteine-674 of the Sarco/endoplasmic Reticulum Calcium ATPase Is Required for the Inhibition of Cell Migration by Nitric Oxide. Atvb 27, 783–790. 10.1161/01.atv.0000258413.72747.23 17234728

[B57] Zweier J. L. Chen C.-A. Druhan L. J. (2011). S-glutathionylation Reshapes Our Understanding of Endothelial Nitric Oxide Synthase Uncoupling and Nitric Oxide/reactive Oxygen Species-Mediated Signaling. Antioxid. Redox Signaling 14, 1769–1775. 10.1089/ars.2011.3904 PMC3078498 21261471

